# Experiences of medical students and faculty regarding the use of long case as a formative assessment method at a tertiary care teaching hospital in a low resource setting: a qualitative study

**DOI:** 10.1186/s12909-024-05589-7

**Published:** 2024-06-05

**Authors:** Jacob Kumakech, Ian Guyton Munabi, Aloysius Gonzaga Mubuuke, Sarah Kiguli

**Affiliations:** 1 School of Medicine, Department of Paediatrics & Child Health, Makerere University, Kampala, Uganda; 2 School of Biomedical Sciences, Department of Anatomy, Makerere University, Kampala, Uganda; 3 School of Medicine, Department of Radiology, Makerere University, Kampala, Uganda; 4 School of Medicine, Department of Pediatrics & Child Health, Makerere University, Kampala, Uganda

**Keywords:** Long case, Formative assessment, Medical education, Low-resource setting, Uganda

## Abstract

**Introduction:**

The long case is used to assess medical students’ proficiency in performing clinical tasks. As a formative assessment, the purpose is to offer feedback on performance, aiming to enhance and expedite clinical learning. The long case stands out as one of the primary formative assessment methods for clinical clerkship in low-resource settings but has received little attention in the literature.

**Objective:**

To explore the experiences of medical students and faculty regarding the use of the Long Case Study as a formative assessment method at a tertiary care teaching hospital in a low-resource setting.

**Methodology:**

A qualitative study design was used. The study was conducted at Makerere University, a low-resource setting. The study participants were third- and fifth-year medical students as well as lecturers. Purposive sampling was utilized to recruit participants. Data collection comprised six Focus Group Discussions with students and five Key Informant Interviews with lecturers. The qualitative data were analyzed by inductive thematic analysis.

**Results:**

Three themes emerged from the study: ward placement, case presentation, and case assessment and feedback. The findings revealed that students conduct their long cases at patients’ bedside within specific wards/units assigned for the entire clerkship. Effective supervision, feedback, and marks were highlighted as crucial practices that positively impact the learning process. However, challenges such as insufficient orientation to the long case, the super-specialization of the hospital wards, pressure to hunt for marks, and inadequate feedback practices were identified.

**Conclusion:**

The long case offers students exposure to real patients in a clinical setting. However, in tertiary care teaching hospitals, it’s crucial to ensure proper design and implementation of this practice to enable students’ exposure to a variety of cases. Adequate and effective supervision and feedback create valuable opportunities for each learner to present cases and receive corrections.

## Background

The long case serves as an authentic assessment method for evaluating medical students’ competence in clinical tasks [1]. This form of assessment requires students to independently spend time with patients taking their medical history, conducting physical examinations, and formulating diagnosis and management plans. Subsequently, students present their findings to senior clinicians for discussion and questioning [2, 3]. While developed countries increasingly adopt simulation-based assessments for formative evaluation, logistical challenges hinder the widespread use of such methods in developing countries [4]. Consequently, the low-resource countries heavily rely on real patient encounters for formative assessment. The long case is one such method predominantly used as a primary formative assessment method during clinical clerkship and offers a great opportunity for feedback [5]. The assessment grounds students’ learning into practice by providing them with rich opportunities to interact with patients and have the feel of medical practice. The long case thus bridges the gap between theory and practice, immersing students in the real tasks of a physician [1]. The complexity of clinical scenarios and the anxiety associated with patient encounters may not be well replicated in simulation-based assessments because diseases often have atypical presentations not found in textbooks. Assessment methods should thus utilize authentic learning experiences to provide learners with applications of learning that they would expect to encounter in real life [6]. This requires medical education and the curriculum to focus attention on assessment because it plays a significant role in driving learning [7]. The long case thus remains crucial in medical education as one of the best ways of preparing for practice. It exposes the student repeatedly to taking medical history, examining patients, making clinical judgments, deciding treatment plans, and collaborating with senior clinicians.

The long case, however, has faced significant criticism in the medical education literature due to perceived psychometric deficiencies [8–10]. Consequently, many universities have begun to adopt assessment methods that yield more reliable and easily defensible results [2] due to concerns over the low reliability, generalizability, and validity of the long case, coupled with rising litigations and student appeals [11, 12]. Despite these shortcomings, the long case remains an educationally valuable assessment tool that provides diagnostic feedback essential for the learning process during clinical clerkship [13]. Teachers can utilize long-case results to pinpoint neglected areas or teaching deficiencies and align with course outcomes.

However, there is a paucity of research into the long case as a formative assessment tool. A few studies conducted in developed countries highlighted its role in promoting a holistic approach to patient care, fostering students’ clinical skills, and a driving force for students to spend time with patients [2, 13], . There is a notable absence of literature on the use of long case as a formative assessment method in low-resource countries, and no published work is available at Makerere University where it has been used for decades. This underscores the importance of conducting research in this area to provide insight into the effectiveness, challenges, and potentials for improvement. Therefore, this study aimed to investigate the experiences of medical students and faculty regarding the utilization of the long case as a formative assessment method within the context of a tertiary care teaching hospital in a low-resource setting.

## Methodology

### Study design

This was an exploratory qualitative study.

### Study setting

The research was conducted at Makerere University within the Department of Internal Medicine. The Bachelor of Medicine and Bachelor of Surgery (MBChB) degree at Makerere University is a five-year program with the first two years for pre-clinical (biomedical Sciences) course and the last three years dedicated to clinical clerkship. Medical students do Internal Medicine clerkships in third- and fifth-year at the two tertiary teaching hospitals namely; Mulago and Kiruddu National Referral Hospitals. The students are introduced to the long case in third-year as Junior Clerks and later in the fifth-year as Senior Clerks. During clerkship, students are assigned to various medical wards, where they interact with patients, take medical history from them, perform physical examinations, and develop diagnosis and management plans. Subsequently, students present their long cases to lecturers or postgraduate students, often in the presence of their peers, followed by feedback and comprehensive case discussions. Students are afforded ample time to prepare and present their cases during ward rounds, at their discretion. The students are formatively assessed and a mark is awarded on a scale of one to ten in the student’s logbook. Each student is required to make a minimum of ten long cases over the seven weeks of clerkship.

### Study participants

The study participants were third- and fifth-year medical students who had completed junior and senior clerkship respectively, as well as lecturers who possessed at least five years of experience with the long case. The participants were selected through purposive sampling. The sample size for the study was determined by data saturation.

### Data collection

Data were collected through Focus Group Discussions (FGDs) and Key Informant Interviews (KIIs). A total of 36 medical students participated in FGDs, reflecting on their experiences with the long case. Five faculty members participated in individual KIIs. The students were mobilized by their class representative and a brief recruitment presentation was made at the study site while the lecturers were approached via email and telephone invitation.

Six FGDs were conducted, three for junior clerks and three for senior clerks. Each FGD comprised of 5–7 participants with balanced male and female gender representation. Data saturation was achieved by the fifth FGD, at which point no additional new information emerged. A research assistant proficient in qualitative research methods moderated the FGDs. The discussions lasted between 55 min and 1 h 10 min and were audio recorded. The Principal Investigator attended all the FGDs to document interactions and record his perspectives and non-verbal cues of participants.

Semi-structured KIIs were used to collect data from Internal Medicine faculty. Five KIIs were conducted, and data saturation was achieved by the fourth interview, at which point no new theme emerged. The Principal Investigator conducted the KIIs via Zoom. Each interview lasted between 25 and 50 min and all were audio recorded. A research assistant proficient in qualitative methods attended all the Zoom meetings. The data collected were securely stored on a hard drive and Google Drive with password protection to prevent unauthorized access.

### Data analysis

Data analysis was done through inductive thematic analysis method. Following each FGD or KII session, the data collection team listened to the recordings to familiarize themselves with the data and develop general ideas regarding the participants’ perspectives. The data were transcribed verbatim by the researchers to generate text data. Two separate transcripts were generated by the Principal Investigator and a research assistant. The transcripts were then compared and manually reviewed by the research team to compare the accuracy with the audio recordings. After transcript harmonization, data cleaning was done for both FGDs and KIIs transcripts.

The transcribed data from both FGDs and KIIs underwent inductive thematic analysis as aggregated data. This involved initial line-by-line coding, followed by focused coding where the relationships between initial codes were explored and similar codes were grouped. Throughout the analysis, the principle of constant comparison was applied, where emerging codes were compared for similarities and differences.

## Study results

### Socio-demographics

A total of 36 medical students participated in the FGDs, comprising 18 junior clerks and 19 senior clerks. The participants were aged between 21 and 25 years except two participants who were aged above 25 (30 and 36 years old). Among the third-year students, there were 10 male and 9 female participants while the fifth-year student comprised of 8 male and 10 female participants.

Five lecturers participated in the Key Informant Interviews, three of whom were females and two male participants. They were aged between 40 and 50 years, and all had over 10 years of experience with the long case. The faculty members included one consultant physician, one associate professor, two senior lecturers, and one lecturer.

### Themes that emerged

Three themes emerged from the study: ward placement, case presentations, and case assessment and feedback.

**Table:** 

Themes	Codes
Theme 1; ward placement	Allocation to specific ward, specialization of the wards, orientation on the ward, and exposure to other ward
Theme 2; case presentation	Variation in the mode of presentation, limited observation of skills, and unreliable presence of lecturers.
Theme 3; case assessment and feedback	Marks awarded for the long case, case write-up, marks as motivators, pressure to hunt for mark Feedback is given to the student, feedback to the lecturer, limitations of the feedback practice

### Theme 1: Ward placement

The study findings disclosed that medical students are assigned to specific wards for the duration of their clerkship. The specialization of medical wards was found to significantly restrict students’ exposure to limited disease conditions found only in their allocated ward.

*With the super-specialization of the units, there is some bias on what they do learn; if a particular group is rotating on the cardiology unit, they will obviously have a bias to learn the history and physical exam related to cardiovascular disease (KII 1).*

The students, particularly junior clerks, expressed dissatisfaction with the lack of proper and standardized orientation to the long case on the wards. This deficiency led to wastage of time and a feeling of being unwelcome in the clerkship.

*Some orient you when you reach the ward but others you reach and you are supposed to pick up on your own. I expect orientation, then taking data from us, what they expect us to do, and what we expect from them, taking us through the clerkship sessions (FGD 4 Participant 1).*

Students’ exposure to cases in other wards poses significant challenges; the study found that as some lecturers facilitate visits to different wards for scheduled teaching sessions, others don’t, resulting in missed learning opportunities. Additionally, some lecturers leave the burden on students’ personal initiative to explore cases in other wards.

*We actually encourage them to go through the different specialties because when you are faced with a patient, you will not have to choose which one to see and not to see (KII 4).*

*Imagine landing on a stroke patient when you have been in the infectious disease ward or getting a patient with renal condition when you have been in the endocrinology ward can create problems (FGD 6 Participant 3).*

### Theme 2 Case presentation

Medical students present their long case to lecturers and postgraduate students. However, participants revealed variations among lecturers regarding their preferences on how they want students to present their cases. While some prefer to listen to the entire history and examination, others prefer only a summary, and some prefer starting from the diagnosis.

*The practice varies depending on the lecturer, as everyone does it their own way. There are some, who listen to your history, examination, and diagnosis, and then they go into basic discussion of the case; others want only a summary. Some lecturers come and tell you to start straight away from your diagnosis, and then they start treating you backward (FGD 6 Participant 3).*

The students reported limited observation of their skills due a little emphasis placed by examiners on physical examination techniques, as well as not providing the students with the opportunity to propose treatment plans.

*When we are doing these physical examinations on the ward no one is seeing you. You present your physical examination findings, but no one saw how you did it. You may think you are doing the right thing during the ward rotations, but actually your skills are bad (FGD 4 Participant 6).*

*They don’t give us time to propose management plans. The only time they ask for how you manage a patient is during the summative long case, yet during the ward rotation, they were not giving us the freedom to give our opinion on how we would manage the patient.(FGD 2Participant 6).*

Supervision was reportedly dependent on the ward to which the student was allocated. Additionally, the participants believe that the large student-to-lecturer ratio negatively affects the opportunity to present.

*My experience was different in years three and five. In year three, we had a specialist every day on the ward, but in year five, we would have a specialist every other day, sometimes even once a week. When I compare year five with year three, I think I was even a better doctor in year three than right now (FGD 1 Participant 1).*

*Clinical training is like nurturing somebody to behave or conduct themselves in a certain way. Therefore, if the numbers are large, the impacts per person decrease, and the quality decreases (KII 5).*

### Theme C: Case assessment and feedback

The study found that a student’s long case is assessed both during the case presentation on the ward and through the case write-up, with marks awarded accordingly.

*They present to the supervisor and then also write it up, so at a later time you also mark the sheet where they have written up the cases; so they are assessed at presentation and write up (KII 2).*

The mark awarded was reportedly a significant motivator for students to visit wards and clerk patients, but students also believe that the pressure to hunt for marks tends to override the goal of the formative assessment.

*Your goal there is to learn, but most of us go with the goal of getting signatures; signature-based learning. The learning, you realize probably comes on later if you have the individual morale to go and learn (FGD 1 participant 1).*

Feedback is an integral part of any formative assessment. While students receive feedback from lecturers, the participants were concerned about the absence of a formal channel for soliciting feedback from students.

*Of course, teachers provide feedback to students because it is a normal part of teaching. However, it is not a common routine to solicit feedback about how teaching has gone. So maybe that is something that needs to be improved so that we know if we have been effective teachers (KII 3).*

Whereas the feedback intrigues students to read more to compensate for their knowledge gap, they decried several encounters with demeaning, intimidating, insulting, demotivating, and embarrassing feedback from assessors.

*Since we are given a specific target of case presentation we are supposed to make in my training*, *if I make the ten, I wouldn’t want to present again. Why would I receive other negative comments for nothing? They truly have a personality effect on the student, and students feel low self-esteem (FGD 1, Participant 4).*

## Discussion

This study aimed to investigate the experiences of medical students and faculty regarding the use of the long case as a formative assessment method at a tertiary care teaching hospital in a low-resource setting. This qualitative research provides valuable insights into the current practices surrounding the long case as a formative assessment method in such a setting.

The study highlighted the patient bedside as the primary learning environment for medical students. Bedside teaching plays a crucial role in fostering the development of skills such as history-taking and physical examination, as well as modeling professional behaviors and directly observing learners [14, 15]. However, the specialization of wards in tertiary hospitals means that students may not be exposed to certain conditions found in other wards. This lack of exposure can lead to issues of case specificity, which has been reported in various literature as a cause of low reliability and generalizability of the long case [16, 17]. Participants in the study expressed feeling like pseudo-specialists based on their ward allocations. This is partly attributed to missing scheduled teachings and poor management of opportunities to clerk and present patients on other wards. Addressing these challenges is essential for enhancing the effectiveness of the long case as a formative assessment method in medical education.

Proper orientation at the beginning of a clerkship is crucial for clarifying the structure and organization, defining students’ roles, and providing insights into clinical supervisors’ perspectives [18]. However, the study revealed that orientation into the long case was unsatisfactory, resulting in time wastage and potentially hindering learning. Effective orientation requires dedicated time and should involve defining expectations and goals, as well as guiding students through the steps of history-taking and physical examination during the initial weeks of the rotation. Contrary to this ideal approach, the medical students reported being taken through systemic examinations when the clerkship was nearing its end, highlighting a significant gap in the orientation process. Proper orientation is very important since previous studies have also documented the positive impact of orientation on student performance [19]. Therefore, addressing the shortcomings in orientation practices identified in this study is essential for optimizing learning outcomes and ensuring that students are adequately prepared to engage in the long case.

There was reportedly a significant variation in the way students present their long cases, with some lecturers preferring only a case summary, while others expect a complete presentation or begin with a diagnosis. While this diversity in learning styles may expose students to both familiar and unfamiliar approaches, providing a balance of comfort and tension [20], it’s essential for students to first be exposed to familiar methods before transitioning to less familiar ones to expand their ability to use diverse learning styles. The variation observed in this context may be attributed to time constraints, as lecturers may aim to accommodate the large number of students within the available time. Additionally, a lack of standardized practices could also contribute to this variation. Therefore, there is a pressing need for standardized long-case practices to ensure a consistent experience for students and to meet the desired goals of the assessment. Standardizing the long case practice would not only provide a uniform experience for students but also enhance the reliability, validity, and perception of fairness of the assessment [9, 21]. It would ensure that all students are evaluated using the same criteria, reducing potential biases and disparities in grading. Additionally, standardized practices facilitate better alignment with learning objectives and promote more effective feedback mechanisms [22].

Related to the above, students reported limited observation of skills and little emphasis placed on them to learn physical examination techniques. This finding resonates with the research conducted by Abdalla and Shorbagi in 2018, where many students reported a lack of observation during history-taking and physical examination [23]. The importance of observation is underscored by the fact that students often avoid conducting physical examinations, as highlighted in Pavlakis & Laurent’s study among postgraduate trainees in 2001 [24]. This study sheds more light on the critical role of observation in forcing medical students to master clinical assessment and practical skills. The study also uncovered that students are rarely given the opportunity to propose management plans during case presentations, which hampers their confidence and learning of clinical decision-making. These findings likely stem from the large student-to-lecturer ratio and little attention given to these aspects of the long case during the planning of the assessment method. The result is students not receiving the necessary guidance and support to develop their clinical and decision-making skills. Therefore, addressing these issues by putting more emphasis on observation of student-patient interaction, management plan, and having a smaller student group is vital to ensure that medical students receive comprehensive training and are adequately prepared for their future roles as physicians.

The study found that the marks awarded for the long case serve as the primary motivator for students. This finding aligns with previous research indicating that the knowledge that each long case is part of assessment drives students to perform their duties diligently [2, 25]. It underscores the crucial role that assessment plays in driving learning processes. However, the pressures to obtain marks and signatures reportedly hinder students’ engagement in learning. This could be attributed to instances where some lecturers relax on supervision or are absent, leaving students to struggle to find someone to assess them. Inadequate supervision by attending physicians has been identified in prior studies as one of the causes of insufficient clinical experience [26], something that need to be dealt with diligently. While the marks awarded are a motivating factor, it is essential to understand other underlying motivations of medical students to engage in the long case and their impact on the learning process.

Feedback is crucial for the long case to fulfill its role as an assessment for learning. The study participants reported that feedback is provided promptly as students present their cases. This immediate feedback is essential for identifying errors and learning appropriate skills to enhance subsequent performance. However, the feedback process appears to be unilateral, with students receiving feedback from lecturers but lacking a structured mechanism for providing feedback themselves. One reason for the lack of student feedback may be a perceived intimidating approach from lecturers which discourages students from offering their input. It is thus important to establish a conducive environment where students feel comfortable providing feedback without fear of negative repercussions. The study underscores the significance of feedback from students in improving the learning process. This aligns with the findings of Hattie and Timperley (2007), who emphasized that feedback received from learners contributes significantly to improvements in student learning [27]. Therefore, it is essential to implement strategies to encourage and facilitate bidirectional feedback between students and lecturers in the context of the long case assessment. This could involve creating formal channels for students to provide feedback anonymously or in a structured format, fostering open communication, and addressing any perceived barriers to feedback exchange [28]. By promoting a culture of feedback reciprocity, educators can enhance the effectiveness of the long case as an assessment tool.

## Conclusions

In conclusion, the long case remains a cornerstone of formative assessment during clerkship in many medical schools, particularly in low-resource countries. However, its effectiveness is challenged by limitations such as case specificity in tertiary care hospitals, which can affect the assessment’s reliability and generalizability. The practice of awarding marks in formative assessment serves as a strong motivator for students but also creates tension, especially when there is inadequate contact with lecturers. This can lead to a focus on hunting for marks at the expense of genuine learning. Thus adequate supervision and feedback practices are vital for ensuring the success of the long case as an assessment for learning.

Furthermore, there is a need to foster standardized long case practice to ensure that scheduled learning activities are completed and that all students clerk and present patients with different conditions from various wards. This will promote accountability among both lecturers and students and ensure a consistent and uniform experience with the long case as an assessment for learning, regardless of the ward a student is assigned.

## Data Availability

The data supporting the study results of this article can be accessed from the Makerere University repository, titled “Perceptions of Medical Students and Lecturers of the Long Case Practices as Formative Assessment in Internal Medicine Clerkship at Makerere University,” available on DSpace. The identifier is http://hdl.handle.net/10570/13032. Additionally, the raw data are securely stored with the researchers in Google Drive.
